# Recycling of Lead-Acid Battery Electrodes Using Sb_2_O_3_ and CuO: Characterization and Electrochemical Investigations

**DOI:** 10.3390/ma18050935

**Published:** 2025-02-21

**Authors:** Delia N. Piscoiu, Simona Rada, Sergiu Macavei, Lucian Barbu, Ramona Suciu, Eugen Culea

**Affiliations:** 1 Faculty of Materials and Environmental Engineering, Technical University of Cluj-Napoca, 400641 Cluj-Napoca, Romaniaeugen.culea@phys.utcluj.ro (E.C.); 2 National Institute for Research and Development of Isotopic and Molecular Technologies, 400293 Cluj-Napoca, Romania; sergiu.macavei@itim-cj.ro (S.M.); lucian.barbu@itim-cj.ro (L.B.); ramona.suciu@itim-cj.ro (R.S.)

**Keywords:** CuO-Sb_2_O_3_-PbO_2_-Pb, spent plates, lead-acid battery, spectroscopic, voltammetric

## Abstract

The recycling of spent automotive batteries is essential for minimizing their environmental impact. This requires eco-innovative methods with low cost and energy use. The present study explores the recycling of battery electrodes through the melt quenching method, a process that incorporates spent anode and cathode plates into a vitreous host matrix. Samples with the xCuO·10Sb_2_O_3_·(90 − x)[4PbO_2_·Pb] composition, where x = 0 to 30 mol% CuO, were prepared by the melt quenching method. The XRD analysis indicates the vitroceramic structures of the obtained samples. Thus, the presence of varied crystalline phases such as Pb_2_(SO_4_)O, PbSO_4_, and metallic Pb was detected. The SEM micrographs highlighted heterogeneous regions within the samples and showed a decreases of the size of crystallites with increased dopant concentrations. IR and UV-Vis spectra suggest that the copper ions act as network modifiers, creating bond defects and free oxygen ions, and yielding a reduction of the optical bandgap energy at higher dopant contents. EPR data show that the shape of the resonance lines and the coordination geometry of the Cu^2+^ ions are influenced by the dopant concentrations. The analysis of the voltammetric data indicates that doping the recycled material with 20 mol% CuO and 10 mol% Sb_2_O_3_ eliminates the process of hydrogen evolution and reduces the anodic electrode passivation.

## 1. Introduction

The recycling of spent lead-acid batteries is essential due to the environmental and health risks associated with improper disposal [1]. A spent plate refers to materials that have exhausted their initial functionality or performance. In this context, electrodes form spent automotive batteries denote used battery electrodes that are no longer operational.

Traditional pyrometallurgical methods for lead recovery from these batteries consume considerable energy and generate significant quantities of polluting products, including sulfur dioxide, lead vapor, and dust [1]. As a result, researchers are exploring alternative recycling techniques, notably hydrometallurgical processes, in order to recover lead and other valuable materials in a more environmentally sustainable manner [1].

A promising method that aims to the effective recovery of lead and of other components of spent lead-acid batteries by reducing their environmental impact is melt quenching. This technique involves melting battery components with a vitreous material at high temperatures to facilitate the separation and recovery of various materials. Notably, this method mitigates hydrogen evolution reactions and partially desulfates both the anodic and cathodic plates [2]. Different additives were tested to enhance the recycling process. The incorporation of CuO and Sb_2_O_3_ into the battery materials was studied in order to observe possible improvements of the properties and the performance of the recovered products [3,4,5]. The composition of the host matrix used for the recycling process plays a crucial role because it influences the overall efficiency and environmental impact of the method. These experimental findings suggest that the use of appropriate additives can optimize synthesis conditions and improve the properties of the resulting materials.

The environmental, health, and economic challenges associated with lead sulfates from spent lead-acid batteries highlight the necessity of their removal for effective recycling. Hydrometallurgical processes proved to be an efficient method for lead recovery because they offer a reduced environmental impact in comparison to the traditional pyrometallurgical methods. Lead-acid batteries consist of lead (Pb) as the anode electrode and lead dioxide (PbO_2_) as the cathode, which are usually immersed in a dilute sulfuric acid electrolyte [6]. It is crucial to recover the spent lead electrodes of batteries using environmentally friendly methods due to their high toxicity [7].

In recent years, the recycling rates for lead batteries have exceeded 95% in Europe, North America, and Japan, with global recycling rates continuing to rise. The primary stages of lead battery recycling entail acid removal, plastic component separation, metallic part processing, and battery paste recycling. Both pyrometallurgical and hydro-electrometallurgical techniques have been employed for the processing of battery pastes [8].

Lead-acid batteries remain widely used, especially due to their high voltage output, reversibility of electrochemical reactions, and reduced incidence of secondary reactions. With ongoing advancements and research, lead-acid batteries continue to serve as a crucial energy source as we move further into the 21st century [9].

Worldwide, over 80% of total lead production is dedicated to the manufacturing of automotive batteries [10]. Lead oxides, particularly PbO and/or PbO_2_, account for up to 50% of the mass of automotive battery paste, with PbSO_4_ comprising the remaining 50% [8]. Despite numerous proposed lead recycling techniques, notable challenges persist, including the complexity and toxicity of the methods, low solubility of lead compounds in solvents, insufficient efficiency in desulfatization, and difficulties associated with the conversion of these compounds back into metal oxides.

This study proposes the recycling of active materials from the anode (Pb/PbSO_4_) and cathode (PbO_2_/PbSO_4_) plates of spent automotive batteries by their introduction into a glass and/or vitroceramic host matrix doped with CuO and Sb_2_O_3_ to enhance the electrochemical and mechanical properties of the final product.

Therefore, the use of specific additives, such as antimony oxide and copper oxide, is essential to stabilize the process and ensures high-quality recyclable products. This strategy effectively controls toxic emissions, stabilizes lead compounds, and contributes to the production of higher-quality recycled materials, thus reducing the environmental impact of battery recycling operations. Antimony trioxide (Sb_2_O_3_) exhibits characteristics that make it a valuable additive to enhance mechanical properties and to remove the passivation phenomena. Passivation refers to the formation of a protective layer on the surface of an electrode, which inhibits electrochemical reactions and reduces its performance. In the context of this article, passivation can negatively impact the efficiency of the electrode by limiting active sites for reaction, increasing resistance, and thereby lowering the overall electrochemical performance of the system. Addressing or mitigating passivation is critical to maintaining the functionality and durability of the electrodes.

The stabilizing capabilities and flame-retardant properties contribute to the effective management of the chemical reactions and the safety of the overall recycling operation [11,12]. These properties are essential in recycling processes since they help to prevent uncontrolled reactions and emissions, ensuring a more efficient and a safer recycling process [5]. As a flame-retardant additive, antimony trioxide (Sb_2_O_3_) restricts the emission of harmful lead vapors, mitigating the environmental impact of the recycling process. The ability of Sb_2_O_3_ to suppress the release of harmful lead emissions is crucial in mitigating the environmental damage associated with the recycling of spent lead-acid batteries [13].

Copper oxide is another additive that plays a crucial role in the melt quenching method due to its unique oxidizing properties and ability to effectively interact with other metal oxides [14]. By enhancing the leaching and oxidation of copper during the recycling process, CuO helps to improve the overall efficiency and quality of the recovered materials [15]. In the context of the melt quenching method and automotive battery recycling, CuO demonstrates particular significance through its contributions in three key areas: thermal stabilization, compatibility with Sb_2_O_3_, and the improvement of electrochemical properties.

CuO helps to stabilize the structure of lead compounds minimizing the risk of volatilization at lower temperatures. This characteristic is crucial for ensuring both the safety and efficiency of the process [14]. The interaction between CuO and Sb_2_O_3_ creates a synergistic effect that enhances the reactivity and stability of chemical processes in battery recycling. This compatibility improves the efficiency and safety of recycling operations, thereby supporting greater sustainability in the process [16]. The addition of CuO contributes to improve electrochemical properties, further optimizing the performance of the material.

Extensive research has examined the prominent role of copper oxide in recycling processes. Integrated recovery methods for copper anode slime from electronic waste smelting have shown the effective utilization of CuO in extracting and purifying valuable metals [17]. Similarly, studies on the leaching of copper metal in sulfuric acid solutions have revealed that the combined use of oxygen and CuO can accelerate the leaching process, thus enhancing its efficiency [14]. Furthermore, the high photoelectrochemical activity of CuO nanoflakes has been explored, highlighting its potential applications in various energy-related domains, including battery recycling [18].

The objective of this study is to optimize the structural, optical, and electrochemical properties of glass–ceramic materials doped with CuO and Sb_2_O_3_. The simultaneous adding of Sb_2_O_3_ and CuO enhance the mechanical and conductive properties of the recycled material. 

## 2. Experimental Procedure

### 2.1. Materials

Samples with the xCuO·10Sb_2_O_3_·(90 − x)[4PbO_2_·Pb] composition, where x = 0, 5, 10, 15, 20, and 30% CuO were synthesized by the melt quenching method utilizing spent plates from automotive batteries as the lead and lead dioxide sources. Additionally, laboratory reagents, namely antimony trioxide (Sb_2_O_3_) and copper oxide (CuO) powders (Sigma-Aldrich, St. Louis, MO, USA), were incorporated into the synthesis process.

The raw materials were accurately weighed to four decimal places (0.0001 g) using an analytical balance. The mixtures of substances were ground and melted in sintered alumina crucibles within an electric furnace that was maintained at a constant temperature of 1050 °C. After 10 min, the samples were rapidly cooled on a stainless-steel plate.

The obtained samples had the appearance of a vitroceramic material. By increasing the copper (II) oxide content in the host matrix, the color of the samples changed from yellow-reddish to brownish-brown.

### 2.2. Methods

The characterization of the obtained materials was performed by X-ray diffraction (XRD) and infrared (IR), UV-Vis, photoluminescence (PL), and electron paramagnetic resonance (EPR) spectroscopies. The electrochemical performances of the recycled materials were characterized by cyclic voltammetry (CV) and linear sweep voltammetry (LSV).

The crystalline or amorphous characteristics of the synthesized samples were analyzed via X-ray diffraction using a Rigaku diffractometer equipped with a graphite monochromator and a copper anode tube (λ = 1.54 Å). The characterization of the samples was completed using scanning electron microscopy with a JEOL-JSM 5510LV Microscope (Tokyo, Japan).

Infrared (IR) spectra were recorded at room temperature using a JASCO 6200 FTIR spectrometer (Tokyo, Japan).

UV-VIS spectra were obtained using a Perkin-Elmer Lambda 45 UV-VIS spectrometer (Waltham, MA, USA) fitted with an integrating sphere, having a wavelength accuracy of ±2 nm.

Electron paramagnetic resonance (EPR) measurements were performed in the X-band frequency range (9.52 GHz) using a Bruker ELEXSYS 500 spectrometer (Billerica, MA, USA).

Photoluminescence (PL) spectra were acquired with an ABLE & Jasco V 6500 spectrometer (Kraków, Poland), utilizing a 150 W xenon lamp.

The cyclic voltammetry (CV) and the linear sweep voltammetry (LSV) measurements were conducted using an Autolab PGSTAT 302N potentioastat/galvanostat (EcoChemie, Ultrecht, The Netherlands) equipped with NOVA 1.11 software. In the three-electrode electrochemical cell, the working electrode was the recycled material doped with antimony and copper, the reference electrode was a Hg/Hg_2_Cl_2_/KCl calomel electrode, and platinum served as counter electrode. A 5 M sulfuric acid solution was selected as the electrolyte to simulate the specific conditions of a car battery.

## 3. Results and Discussion

### 3.1. Structural Characterization from the Analysis of X-Ray Diffraction (XRD) and Scanning Electron Microscopy (SEM)

**Figure 1:**
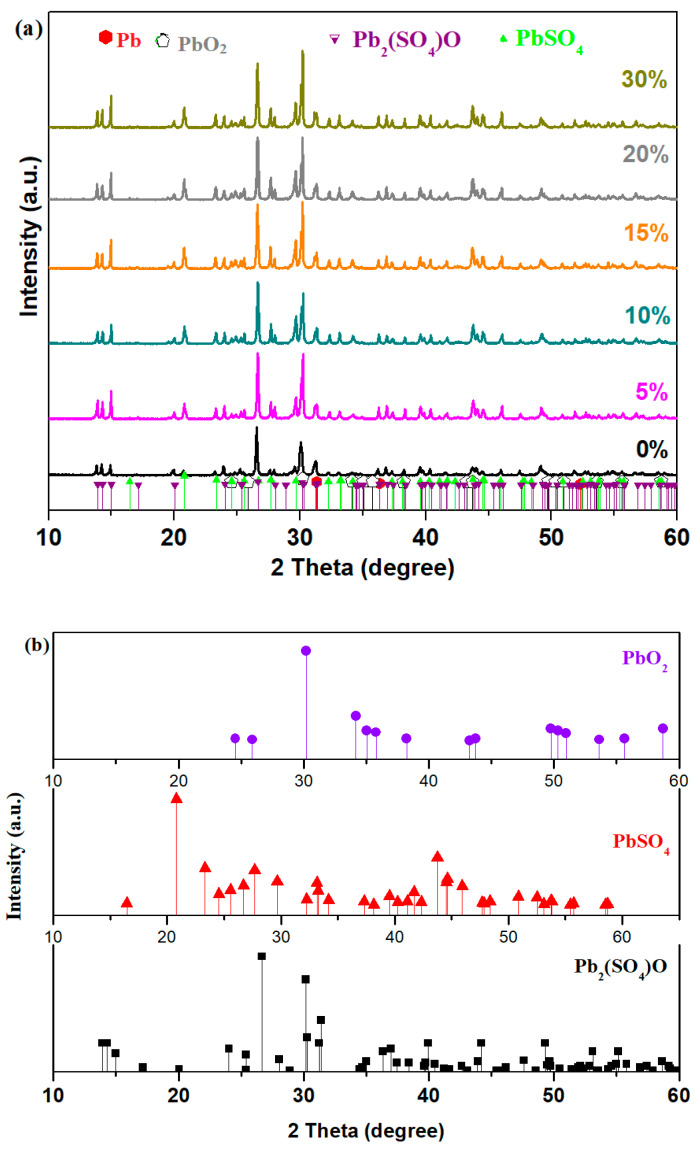
(**a**) X-ray diffraction (XRD) patterns of the recycled samples and (**b**) the index of the phase peaks for Pb_2_(SO_4_)O, PbSO_4_, and PbO_2_ crystalline phases according to standard PDF data.

Figure 1 shows the X-ray diffraction (XRD) patterns for the obtained samples with the xCuO·10Sb_2_O_3_·(90 − x)[4PbO_2_·Pb] composition, where x varies between 0 and 30% CuO. The XRD analysis reveals a vitroceramic structure consisting of four crystalline phases: the Pb_2_(SO_4_)O = PbO·PbSO_4_ = Pb_2_SO_5_ phase with a monoclinic structure, the PbSO_4_ phase with an ortho-rhombic structure, the PbO_2_ phase with orthorhombic structure, and metallic Pb with a cubic structure. The term vitroceramic or glass–ceramic refers to materials that combine the properties of glasses (amorphous) and ceramics (crystalline), consisting of a glassy matrix with dispersed crystalline phases.

The intensities of the diffraction peaks situated at about 26.6° (of 100% intensity) and 30.16° (of 74.3% intensity), corresponding to the Pb_2_SO_5_ crystalline phase, overlapped with the diffraction peak of 100% intensity at 30.2° of the PbO_2_ crystalline phase, show some modifications by doping with higher CuO content. For the samples with 5, 15, and 20% CuO, both peaks have same intensities suggesting that the amount of PbO_2_ crystalline phase increased. This observation can be also evidenced for the sample with 30% CuO when the intensity of the second peak was enhanced. An opposite effect can be observed in the sample with 10% CuO because the second diffraction peak was decreased.

In brief, by adding higher CuO levels into the host matrix, the amount of the oxo-sulfated phase decreases while the content of PbO_2_ crystalline phase is enriched.

**Figure 2:**
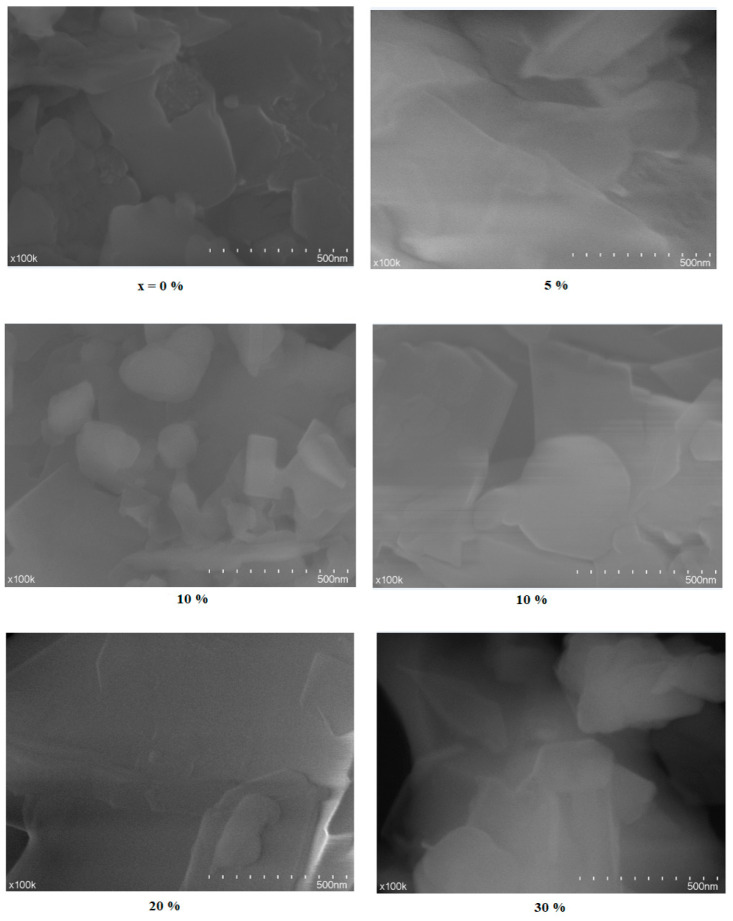
SEM micrographs of the recycled system at magnification of ×100k.

SEM micrographs of the studied samples are presented in Figure 2. At concentrations between 0% and 5%, the structure exhibits an amorphous organization without significant variations. Details about the microstructure or local defects are not evident in these regions.

For the 10% sample, the structure starts to show more pronounced variations, with separations between the layers and amorphous regions. A certain granular distribution and variation in layer thickness can be observed, which may affect the macrostructural order. Possible local accumulations or small pores become more evident at this concentration.

For the 20% sample, clearly separated regions are observed, with more irregular edges and differences in contrast. The presence of discontinuities indicates a loss of structural uniformity at a larger scale. The visible irregularities suggest the presence of local defects or more pronounced microstructural variations.

In the 30% sample, the structure becomes visibly more disordered, with irregular boundaries and accumulations of granular material. The material becomes completely disordered on a larger scale, suggesting a total loss of macrostructural order. The granular texture can be associated with local defects, porosity, or nanometric - level segregations.

Structures are more homogeneous at lower concentrations and become increasingly disordered as the concentration increases, reflecting the loss of long-range order and the accentuation of short-range defects.

### 3.2. Infrared (IR) Spectra

**Figure 3:**
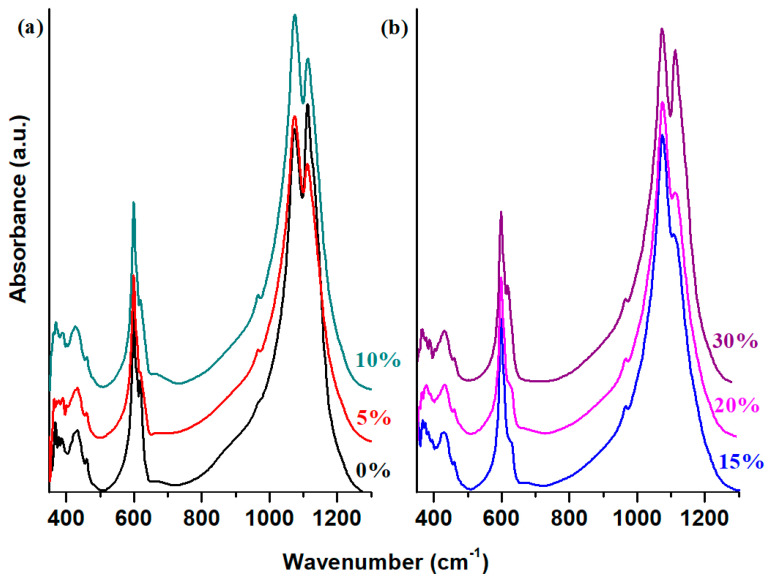
The IR spectra of the studied system with the xCuO·10Sb_2_O_3_·(90 − x)[4PbO_2_·Pb] composition with (**a**) x = 0, 5, 10% CuO and (**b**) x = 15, 20, and 30% CuO recorded in the region between 350–1300 cm^−1^.

The FTIR spectra of the recycled and copper–antimony doped system are illustrated in Figure 3. The assignments of the IR bands are listed in Table 1. The IR band centered at about 465 cm^−1^ is attributed to the stretching vibrations of the Pb-O bond within the [PbO_4_] structural units [19]. The intensity of this band increases for the samples with 15 and 20% CuO. This shows an increase of the polymerization degree of the vitreous network due to the increased amount of [PbO_4_] structural units. For other samples, the intensity of this band decreases due to the conversion of [PbO_4_] structural units in [PbO_3_] structural units.

The IR bands observed at 600, 1075, and 1110 cm^−1^ correspond to sulfate ions [20]. The IR band at 600 cm^−1^ is also attributed to the bending vibrations of the Sb-O-Sb angles within the [SbO_3_] structural units. The intensity of the IR band at about 600 cm^−1^ decreases at higher concentrations of the dopant up to 30 mol% CuO, indicating that the copper oxide induces distortions in the Sb-O-Sb angles. A decreasing trend in their intensities can be observed at an elevated dopant level up to 30 mol%. The intensities of the last two IR bands, 1075 and 1110 cm^−1^, decreases and attains a minimum value for the sample with 20% CuO.

The IR band centered at about 620 cm^−1^ can be assigned to the contributions of the S-O bonds from the sulfite units, overlapped with the contributions of stretching vibrations of the Cu-O bonds from [CuO_4_] structural units. By doping, the intensity of the band changes due to the lattice adjustments accommodating excess oxygen. For the samples with 15 and 20%, the intensity of this IR band decreases drastically up to its disappearance. After that, the intensity of the mentioned band increases again for 30 mol% CuO.

The IR band of lower intensity localized at 670 cm^−1^ increases in intensity with the addition of CuO content up to 20 mol%, followed by a decline for the sample with 30 mol% CuO. This trend indicates a polymerization process of the host matrix by the formation of [PbO_n_] structural units (particularly [PbO_4_] ones) along with a reduction process of the sulfate units in the host matrix.

The regions of the IR bands with high intensity, situated between 900 and 1200 cm^−1^, correspond to contributions of the sulfate units, overlapped with those of the stretching vibrations of the Pb-O bond in [PbO_n_] units with n = 3 and n = 4. A decreasing trend in the intensity of these bands is observed for higher CuO levels.

Furthermore, the analysis of the IR spectra revealed significant structural changes as the dopant concentration increased, particularly in the intensity variations at 620, 1075, and 1100 cm^−1^.

These findings suggest two mechanisms that occur in the host matrix by adding of CuO contents in the host matrix: (i) by adding 5 ≥ x ≤ 20% CuO, an adjusted process of the excess oxygen atoms takes place in the host matrix by the formation of [CuO_n_] and [PbO_n_] structural units (mainly with n = 4), suggesting an increase in the disorder degree, and (ii) at a higher CuO level up to 30%, the conversion of [PbO_4_] into [PbO_3_] structural units reflects that the internal structure of the host matrix is rearranged according to the SEM micrographs, and the amount of PbO_2_ crystalline phase was also increased.

### 3.3. Ultraviolet–Visible (UV-Vis) Spectroscopy: Determination of Optical Band Gap Energy (Eg)

**Figure 4:**
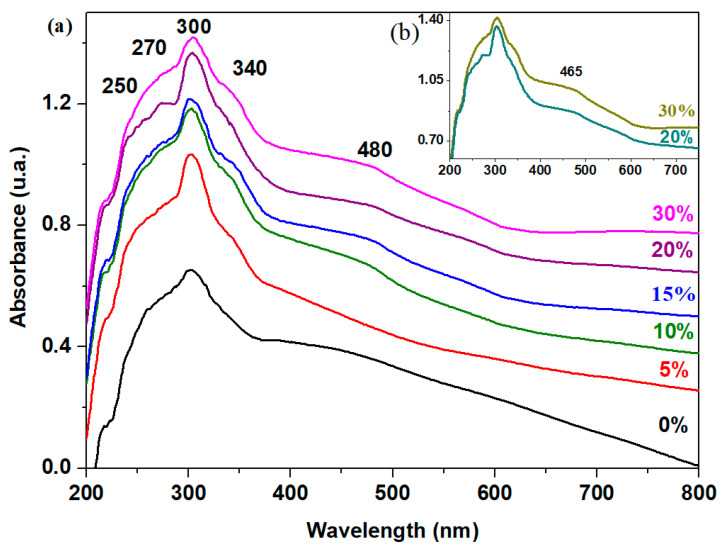
The UV-Vis spectra of studied samples of the xCuO·10Sb_2_O_3_·(90 − x)[4PbO_2_·Pb] composition, where (**a**) x = 0–30% CuO and (**b**) x = 20 and 30% CuO.

The UV-Vis spectra of the recycled system registered for the 200–800 nm region are presented in Figure 4. The analysis of the UV-Vis spectra reveals two groups of absorption bands: (a) some significant absorption bands in the range of 200–350 nm along with (b) absorption bands of lower intensity in the range of 450–800 nm.

The first group of UV-Vis bands shows a band with a maximum intensity around 300 nm, corresponds to electronic transitions of 6s^2^ → 6s^1^6p^1^ type within the host matrix and are due to the Pb^2+^ ions (Table 2). The shoulder centered at 250 nm is associated with the electronic transitions of 3d^10^ → 3d^9^ 4s^1^ type of the Cu^1+^ ions [21]. This feature becomes more prominent and rises in intensity by the addition of higher dopant contents.

For the samples with x ≥ 10 mol% CuO, a new UV-Vis band appears at 465 nm. This band corresponds to charge transfer transitions between Cu^1+^ and Cu^2+^ ions and overlaps the electronic transitions of non-bridging oxygen atoms (at over 420 nm).

A maximum value of intensity of this band occurs for the sample with 30 mol% CuO, indicating a direct relationship between dopant concentration and observed spectroscopic behavior. This trend can be explained considering the conversion of Cu^2+^ → Cu^+^ ions at higher dopant levels that strongly affects the optical properties of the material. In addition, for the sample with 30 mol% CuO, the intensity of the band centered at 740 nm increases suggesting the increase of the electronic transitions of the d-d type of Cu^2+^ ions.

In conclusion, the analysis of the UV-Vis data confirms the presence of Cu^1+^, Cu^2+^, and Pb^2+^ ions in the recycled materials. Higher dopant concentrations yield a conversion of Cu^2+^ ions into Cu^+^ ions.

**Figure 5:**
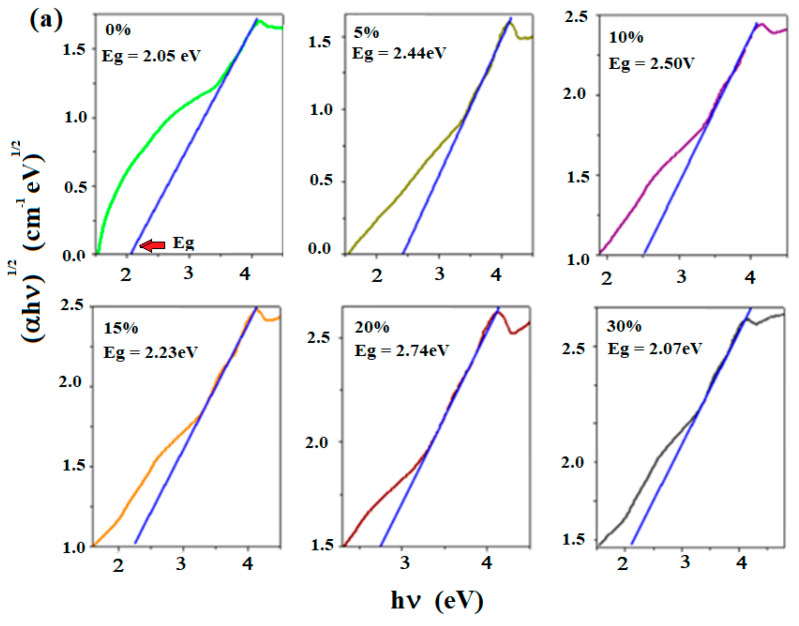

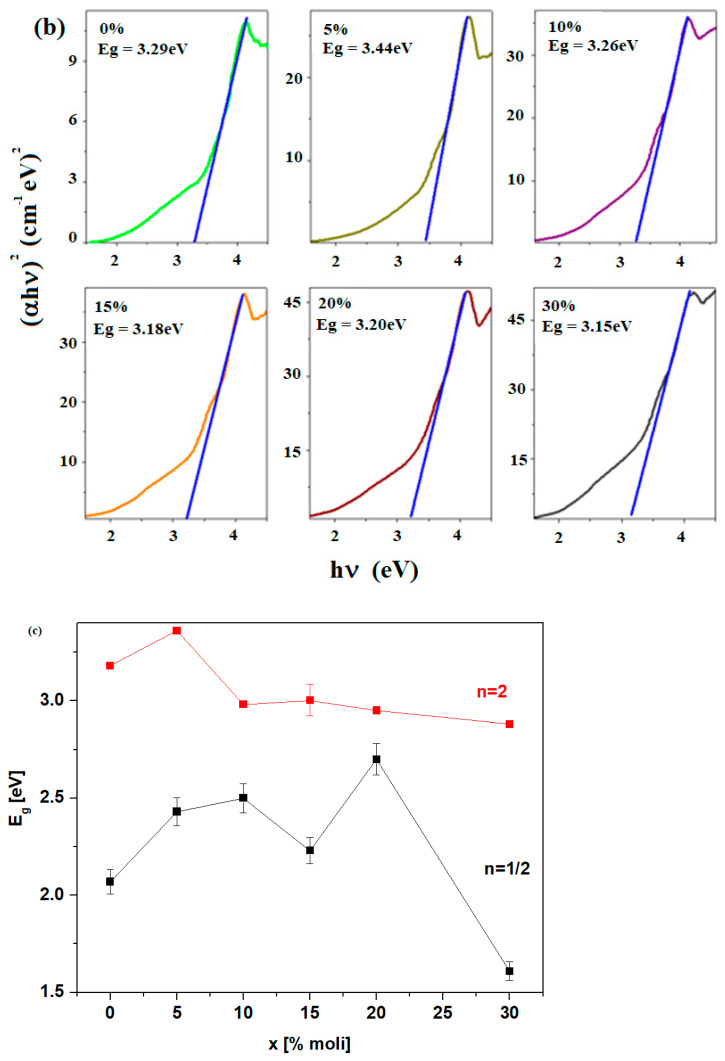
(**a**) Dependence of (αhν)^1/2^ versus photon energy (hν) and (**b**) Dependence of (αhν)^2^ versus hν (the blue line shows the extrapolation of the optical band gap energy, E_g_). (**c**) Compositional evolution of optical gap energy values (E_g_) of the recycled system. Data are shown as a mean of standard deviation (with percent of the data of 3%).

The values of (αhν)^1^/^2^ and (αhν)^2^ for the recycled system were determined from the UV-Vis spectra using their plots versus the photon energy (hν) (shown in Figure 5). The values of the optical band gap energy (E_g_) for the direct transitions (n = 1/2) and indirect transitions (n = 2) were determined by extrapolating the linear region of the (αhν)^1^/^2^ versus hν and (αhν)^2^ versus hν plots to the point where αhν → 0 [19].

For direct transitions, the values of the gap energy are situated between 2.05 eV to 2.74 eV, while a wide band gap energy ranging from 3.15 eV to 3.24 eV was determined for indirect transitions [22,23].

The compositional evolution of the optical band gap energy (E_g_) illustrated in Figure 5c demonstrates a non-linear variation. This non-linearity is attributed to structural changes in the host matrix determined by the CuO doping. The lowest value of the gap energy is observed for the sample with x = 30 mol% CuO, indicating an increased concentration of defects or/and non-bridging oxygen ions.

The gradual addition of copper (II) oxide to the host matrix generates changes in the basic structural units of the lead atoms and links with the non-bridging oxygen ions yielding the formation of lead oxo-sulfate phases, in agreement with the XRD data.

### 3.4. Structural Investigation by Photoluminescence (PL) Spectra

**Figure 6:**
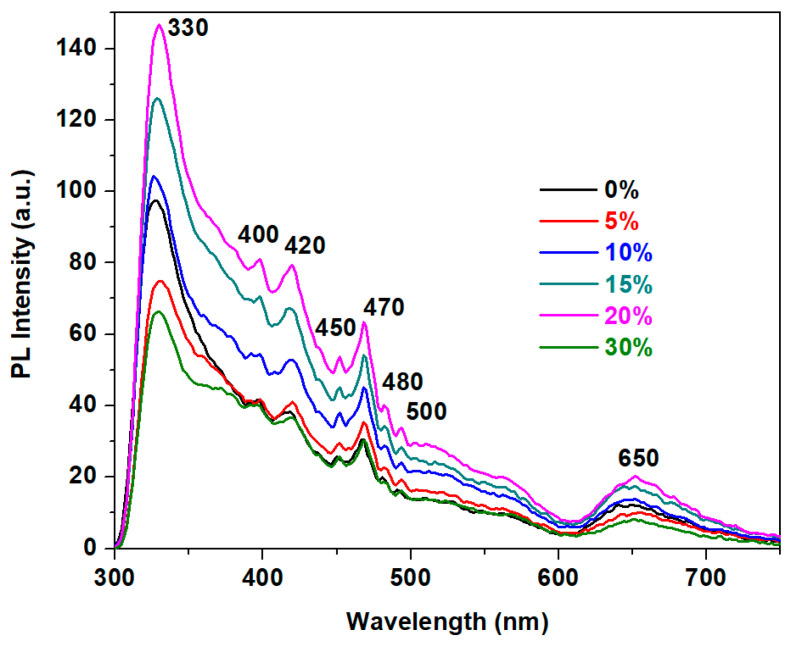
The photoluminescence (PL) spectra of the xCuO·10Sb_2_O_3_·(90 − x)[4PbO_2_·Pb] vitreous system with x = 0–30% CuO.

The photoluminescence (PL) spectra of the studied samples are shown in Figure 6.

The features of the PL spectra demonstrate intense bands centered at 330, 400, 420, and 470 nm along with medium-intensity bands at 450, 480, 500, 560, and 650 nm. The assignment of these PL bands is detailed in Table 3.

The PL bands of the undoped matrix are assigned to the luminescence of Pb^2+^ ions which are not chemically bonded within the matrix. These bands overlap the emission bands due to the surface defects and oxygen vacancies. Thus, the PL bands at 330 and 420 nm are associated with compensated and ionized oxygen vacancies.

With the addition of small amounts of CuO up to x = 5 mol% CuO, the intensity of the PL bands decreases sharply suggesting that the amount of defects in the host matrix increases. At higher dopant levels situated between 10 ≤ x ≥ 20 mol% CuO, an increase in the intensity of all PL bands was observed. This evolution is due to the increase of the number of oxygen vacancies and the accommodation of the matrix with the excess of oxygen atoms.

With a further increase of the dopant level up to x = 30 mol% CuO, the intensity of the PL bands decreases, again indicating the enhancement of the number of defects in the host matrix, the reduction in Pb^2+^ ions, and/or the conversion of Cu^2+^ into Cu^+^ ions.

In summary, the analysis of the PL spectra highlights the following mechanisms:

(i) For smaller dopant amounts up to x ≤ 5 mol% CuO, the excess of oxygen atoms is accommodated with the host matrix by formation of [PbO_n_] structural units. The Cu^2+^ ions occupy the defect positions resulting from Sb-O-Sb or Pb-O-Pb angle deformations, yielding to a decrease in PL bands intensity.
(ii) For the samples with 10 ≥ x ≤ 20 mol% CuO, the PL bands intensities increase indicating a rise of the amount of Pb^2+^ and Cu^2+^ ions in the structure of the host matrix, alongside with a reduction of the number of defects and interstitial sites.
(iii) At higher dopant levels up to 30 mol% CuO, a quenching effect of the luminescence occurs due to the increase of defects and depolymerization process in the host matrix.

Overall, the photoluminescence spectra suggest that the PL intensity increases with CuO concentrations between 10% and 20 mol%, reaching a maximum value at x = 20 mol% CuO. This result implies that the host matrix can accommodate more oxygen vacancies, as well as Pb^2+^, Cu^2+^, and Cu^1+^ ions, in agreement with the SEM data. In contrast, samples with x = 5% and 30 mol% CuO exhibit the quenching of luminescence due to the limited ability of the host network to adjust to the excess of oxygen atoms.

The photoluminescence analysis indicates that increasing CuO concentrations up to 20% enhances luminescence, while for the x = 5% and 30% mol% CuO a quenching of luminescence occurs due to the inability of the matrix to accommodate the excess oxygens and defects.

### 3.5. Electron Paramagnetic Resonance (EPR) Spectra

Electron paramagnetic resonance (EPR) spectroscopy is used to investigate the local structure surrounding paramagnetic centers. Thus, we investigated the microvicinity of the copper ions in the host vitroceramic matrix. The obtained EPR data provide information concerning the distribution of copper ions in locations with different structures and permit to determine the coordination numbers and valence states of the paramagnetic ions [24].

**Figure 7:**
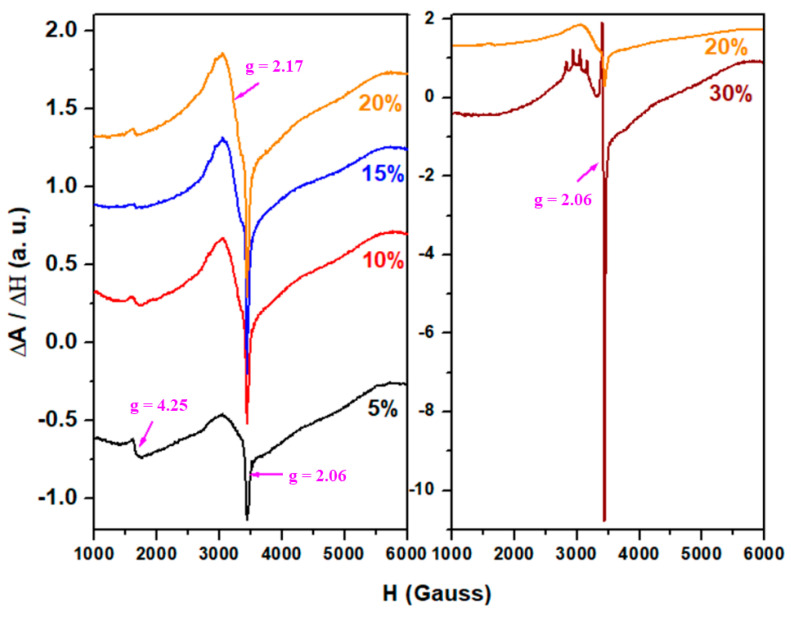
The EPR spectra of the xCuO·10Sb_2_O_3_·(90 − x)[4PbO_2_·Pb] recycled system where x = 5, 10, 15, 20, and 30% CuO.

The EPR spectra of the studied xCuO·10Sb_2_O_3_·(90 − x)[4PbO_2_·Pb] system where x = 5, 10, 15, 20, and 30% CuO, recorded at room temperature, are presented in Figure 7. The predominant paramagnetic ion present in the vitroceramic matrix is the Cu^2+^ ion. Note that the Cu^1+^ ion does not show EPR absorptions.

The analysis of the EPR data suggests that the resonance lines attributed to Cu^2+^ ions are influenced by the concentration of CuO in the host vitroceramic matrix. The Cu^2+^ ions exhibit a hyperfine structure due to the interaction of their nuclear spin (S = 3/2) with the unpaired electron spin. This interaction results in a set of four absorption lines (2S + 1 = 4) corresponding to the magnetic quantum numbers −3/2, −1/2, +1/2, and +3/2 with both parallel (//) and perpendicular (⊥) components. In the case of Cu^2+^ ions located at microvicinities with tetragonal symmetry, EPR spectra with three absorption bands are reported while in the case of a rhombic symmetry, four EPR bands can be observed [25].

The detailed analysis of EPR data indicates two overlapped resonance lines at g ≈ 2.17 and g ≈ 2.06, respectively. The signal centered at g ≈ 2.17 that shows a hyperfine structure is associated with isolated Cu^2+^ ions. For samples with x ≤ 20 mol% CuO, the hyperfine structure is poorly resolved in both the parallel and perpendicular bands. In contrast, for the sample with x = 30 mol% CuO, the four resonance lines in the parallel band are well resolved. The apparition of the hyperfine structure at higher CuO contents up to 30% can be associated with a decrease of the amount of the Cu^+2^ ions due to a Cu^+2^ → Cu^+1^ conversion. The resonance signal localized at g ≈ 2.06 corresponds to the coupled or clustered Cu^2+^ ions.

By increasing the CuO content up to x ≤ 20 mol% CuO, the intensity of the resonance signal with the unresolved hyperfine structure grows. This suggests that the Cu^2+^ ions are mainly a coupled or clustered species alongside a few isolated Cu^2+^ ions.

At higher CuO contents up to 30 mol%, the intensity of the resonance signal increases significantly and the parallel hyperfine components are well-resolved, showing four distinct lines along with a narrow resonance signal of high-intensity centered at g ≈ 2. This structural evolution suggests the presence of isolated Cu^2+^ ions and of some clustered Cu^2+^ ions. This behavior can be attributed to the conversion of some Cu^2+^ → Cu^+^ at higher dopant levels in the host matrix.

Cu^2+^ ions are generally found in octahedral sites corresponding to [CuO_6_] structural units. However, due to the degeneracy of the d_x2-y2_ orbital (unoccupied by electrons), they exhibit tetragonal distortions instead of cubic symmetry [19]. Consequently, in the sample with x = 30 mol% CuO, the Cu^2+^ ions occupies distorted tetragonal or rhombic geometries.

EPR results indicate that the coordination geometry of the Cu^2+^ ions is octahedral with tetragonal distortions. The Cu^2+^ ions are mainly in octahedral sites for the samples with x ≤ 20 mol% CuO, while for the sample with x = 30 mol% CuO, the Cu^2+^ ions exhibit octahedral geometries with tetragonal distortions. Copper ions situated in the distorted octahedral positions contribute to the depolymerization of the host matrix structure generating additional defects and non-bonding oxygen atoms.

The addition of higher CuO concentrations up to 30 mol% causes distortions in the [CuO_6_] octahedral units and facilitates the conversion of Cu^2+^ into Cu^+^ ions. This change is in agreement with a significant decrease of the optical band gap energy and a quenching effect on luminescence in the recycled vitroceramic matrix.

### 3.6. Electrochemical Characterization Using Measurements of Cyclic Voltammetry (CV) and Linear Sweep Voltammetry (LSV)

**Figure 8:**
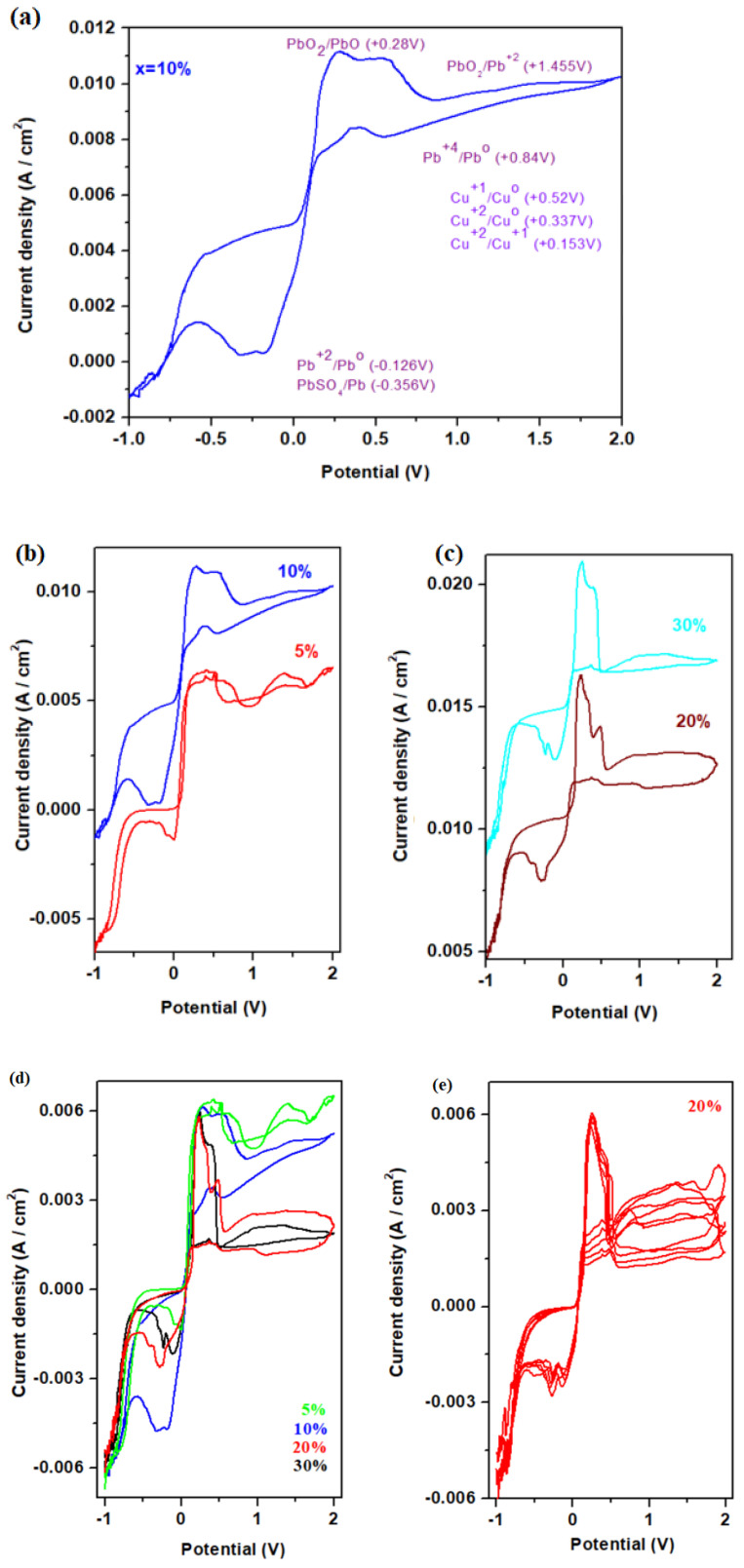
Cyclic voltammogram of the samples with (**a**) 10% CuO, (**b**) 5%, 10%, (**c**) 20%, and 30% CuO, (**d**) 5, 10, 20, and 30% CuO and (**e**) 20% CuO scanned after five cycles as the electrode material in a 5 M sulfuric acid solution using a scan rate of 10 mV/s.

The electrochemical behavior of recycled and copper–antimony doped materials as working electrodes in lead batteries was investigated using cyclic voltammetry [26]. The cyclic voltammogram and features the oxidation and reduction peaks for the electrode material with x = 10% mol CuO are presented in Figure 8a.

The chosen interval allows for the evaluation of the stability of the material at both positive and negative potentials. For example, the 20% concentration exhibits a repeatable evolution of the curves in the positive potential range, indicating good stability in this region. A comparative analysis among different concentrations (5%, 10%, 20%, 30%) within this interval reveals significant variations in current density, suggesting a clear influence of concentration on the electrochemical properties.

At negative potentials (−1 V to 0 V), the currents are low, indicating either a material that is less reactive in this range or a lack of reducible species in the solution. It is important to exclude regions where the material is not active. At positive potentials (0 V to 2 V), the current density increases significantly, especially for the 20% and 30% concentrations. This behavior indicates that the material is efficient in oxidation processes, a valuable characteristic for catalytic applications.

The increase in current with higher concentrations suggests a direct dependency between current density and the proportion of active material, which can justify optimizing the concentration for maximum performance. The chosen interval reflects standard conditions for most electrochemical applications, such as sensors or catalytic devices.

In the positive region of current density two prominent oxidation (anodic) peaks centered at about +0.28 V and +0.52 V, respectively, are observed along with a smaller peak localized at +1.455 V (corresponding to the PbO_2_/Pb^2+^ redox system). The first anodic peak corresponds to the oxidation of PbO to PbO_2_, while the second peak is attributed to the oxidation process of the metallic copper to Cu^+^ and/or Cu^2+^ ions.

In the cathodic region, four distinct peaks are identified at +0.56 V, +0.28 V, −0.17 V, and −0.324 V. The first peak situated at +0.56 V, arises from two overlapping waves situated at +0.84 V for the Pb^4+/^Pb^0^ redox systems and another at +0.52 V for the Cu^+/^Cu redox processes. The peak localized at +0.28 V indicates the reduction processes of Cu^2+^ ions to Cu^+^ ions or Cu^0^. The last peaks situated at −0.17 V and −0.324 V are attributed to the reduction processes of sulfate ions and Pb^2+^ to metallic lead, respectively.

Figure 8 shows cyclic voltammograms for electrode materials with the composition xCuO·10Sb_2_O_3_·(90 − x)[4PbO_2_·Pb], where x = 0, 5, 10, 20, and 30 mol% CuO in a 5 M sulfuric acid solution. The scan rate of the results from the cyclic voltammograms was performed at 10 mV/s. The shapes of the CV curves registered for the varied CuO concentrations are not similar and do not represent a single redox reaction taking place at the sample electrode.

A comparative analysis of these voltammograms reveals that the maximum current density occurs for the electrode with 5 mol% CuO, followed by the electrode with 10 mol% CuO. In contrast, electrodes with 5% and 30 mol% CuO show reactions associated with hydrogen evolution.

Cyclic voltammograms scanned after five cycles for the sample with 20 mol% CuO (Figure 8e) show irreversible processes attributed to the dimerization of the sulfate ions in solution due to the production of the S_2_O_8_^2−^/2SO_4_^2−^ redox systems around 2 V.

In conclusion, the presence of sulfate ions results in a significant irreversibility in the voltammograms, with the highest current density observed at low copper oxide contents (specifically, x = 5% and 10 mol%). The electrodes with x = 10% and x = 20% CuO do not exhibit hydrogen evolution reactions. Thus, the electrode with x = 20% is recommended as the most suitable option for an automotive battery cathode because the amount of PbO_2_ crystalline phase was enriched by CuO doping process. Further verification will be conducted through linear sweep voltammetry measurements.

**Figure 9:**
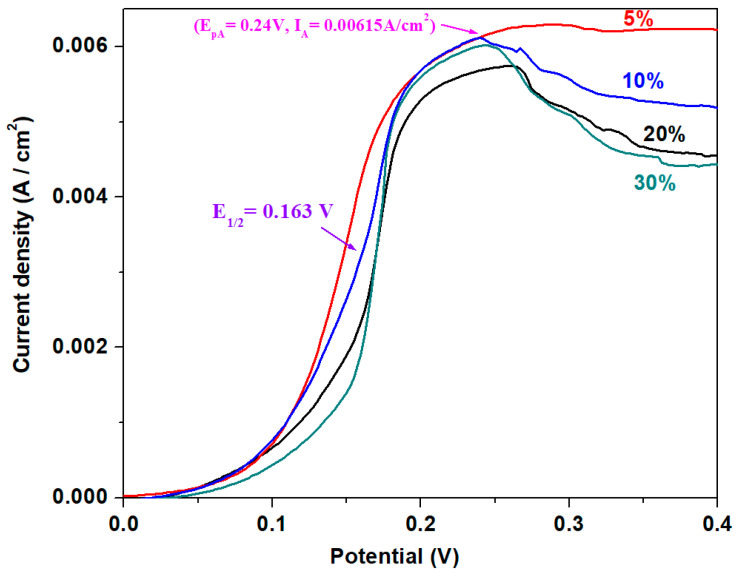
Linearly scanned voltammograms recorded for the electrode with x = 5, 10, 20, and 30% CuO in 5 M sulfuric acid solution at a scan rate of 10 mV/s.

Linear sweep voltammetry enables the comparative identification of the first oxidation peak in the studied system. Figure 9 illustrates linear sweep voltammograms of the studied samples in a 5 M sulfuric acid solution. These measurements provide a detailed view of the oxidation processes, revealing insights into the electrochemical behavior of various CuO concentrations within the recycled matrix [27].

From Figure 9, it is observed that the oxidation peak current is highest for electrodes with x = 5% and x = 10 mol% CuO, while the lowest value is obtained for the electrode with x = 20 mol% CuO.

The electrochemical parameters obtained through cyclic and linear sweep voltammetry after the first cycle of scanning are presented in Table 4. The formal potential, E_f_ depends on the concentration of the active species. From the Figure 8a, the CV profile obtained in 5 M sulfuric acid shows an oxidation peak at 0.27 V and a reduction peak at −0.17 V, with a formal redox potential, E_f_ of 0.05 V and the oxidation and reduction peak potential separation, ΔE of 440 mV. A large potential gap of 50 mV was observed between the oxidation and reduction peaks indicating the kinetics of electron transfer process is very slow in aqueous 5 M H_2_SO_4_. A decrease in the values of the CV peak currents and formal potential were observed for the sample with 20 mol% CuO.

**Figure 10:**
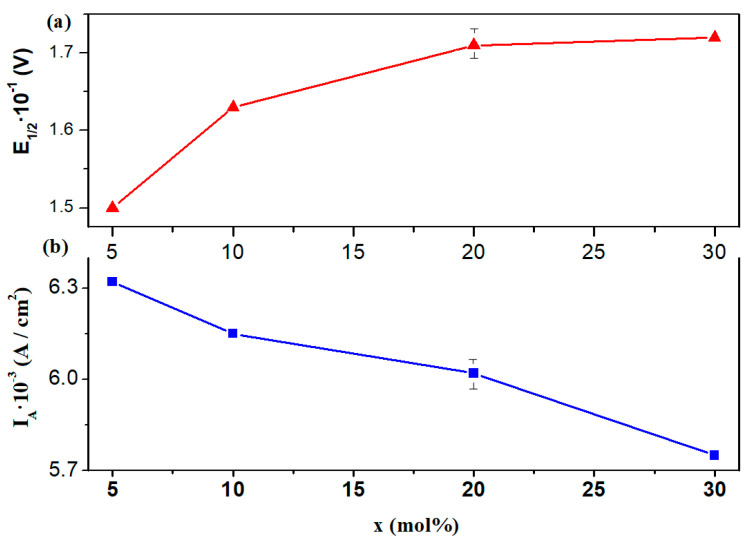
Compositional evolution of electrochemical parameters: (**a**) half-wave potential (E_1/2_) and (**b**) peak current density (I_A_). Data are shown with error bars of 3%.

Figure 10 illustrates the compositional changes of several electrochemical parameters, including the half-wave potential (E_1/2_) and peak current density (I_A_). An almost linear trend in peak current density was noted when increasing the CuO content in the host matrix. When higher CuO concentrations were incorporated into the host matrix, the half-wave potential exhibits a gradual increase up to 30 mol%.

## 4. Conclusions

The prepared vitroceramic samples exhibit a modification of color from reddish-yellow to brownish-brown by increasing of CuO concentration in the host matrix which influences the optical properties of material.

The X-ray diffraction (XRD) analysis proves the presence of four distinct crystalline phases in the glass–ceramic structure, namely the Pb_2_(SO_4_)O, PbSO_4,_ PbO_2_ and Pb.

The SEM micrographs reveal heterogeneous regions with smaller crystallite sizes at higher CuO concentrations.

The incorporation of higher CuO content into the vitreous system significantly affects the intensity of IR bands assigned to the sulfate units and yield the formation of new structural units, such as [CuO_n_] and [PbO_n_]. This structural evolution suggests that copper oxide plays the role of network former at smaller CuO contents while in the case of higher contents behaves as a network modifier agent.

The UV-Vis data reveal the presence of Cu^1+^, Cu^2+^, and Pb^2+^ ions in the recycled materials. As the dopant concentration increases, a conversion of Cu^2+^ ions into Cu^1+^ ions is observed.

The study of the photoluminescence (PL) spectra of the recycled system with varying compositions of CuO reveals a complex relationship between the doping levels and luminescent characteristics. Initial doping with low concentrations of CuO leads to a decrease in the intensity of the PL bands, suggesting an increase of the number of defects in the host matrix. When the CuO concentration rises to between 10% and 20%, the intensity of the PL bands increases indicating the formation of additional oxygen vacancies and favorable bonding interactions (Cu-O and Pb-O-Cu) that produce and enhance the luminescence. At higher concentrations up to 30 mol% CuO, a significant drop in PL intensity occurs, indicating an excessive number of defects, a reduction of the amount of Pb^2+^ ions, and a Cu^2+^ → Cu^1+^ conversion process.

EPR spectra show the resonance lines due to the Cu^2+^ ions situated within the microvicinities with varied coordination geometries. The increase in CuO level in the vitroceramic network affects the shape of the resonance lines.

The cyclic voltammograms of the electrode materials shows irreversible processes due to the dimerization effect of the sulfate ions. The samples with x = 10% and x = 20% CuO do not show hydrogen evolution reactions. The addition of CuO dopant up to 20% also minimizes the passivation phenomenon.

The study recommends the sample with 20% mol CuO as an ideal candidate for cathodic electrodes in batteries due the partial desulfatization of the spent plates, the enhanced stability, the presence of PbO_2_ crystalline phase, and the performance in sulfuric acid environments. This eco-innovative method optimizes recycled electrodes from spent automotive batteries.

## Figures and Tables

**Table 1:** Wavenumbers and assignments of the IR absorption bands of the xCuO·10Sb_2_O_3_·(90 − x)[4PbO_2_·Pb] vitroceramic system with x = 0–30% CuO.

Wavenumbers (cm^−1^)	Assignments of the IR Bands
360–560	Deformation vibrations of Pb-O-Pb and O-Pb-O angles in [PbO_4_] structural units; stretching vibrations of the Cu-O bond.
465	Deformation vibrations of Pb-O-Pb and O-Pb-O angles in [PbO_4_] structural units.
486–604	Sb-O elongation vibrations of Sb-O structural units [SbO_3_].
560–740	Deformation vibrations of Pb-O-Cu and Cu-O- Cu angles.
600	Bending vibrations of Sb-O-Sb angles within the structural units [SbO_3_].
620	Elongation vibrations of Cu-O bond in [CuO_4_] structural units.
650–850	Pb-O bond elongation vibrations in [PbO_n_] structural units.
860	Elongation vibrations of Cu-O bonds in [CuO_6_] structures.
875	Elongation vibrations of the Pb-O bond in [PbO_6_] structural units.
900–1200	Elongation vibrations of the Pb-O bond in [PbO_n_] structural units with n = 3 and 4.
1050, 1150	Vibrations related to sulfate ions.

**Table 2:** Assignments of the UV-VIS bands for the recycled system.

Wavelength (nm)	Assignments of UV-Vis Bands
250	Electronic transitions of Cu^1+^ ions (3d^10^ → 3d^9^ 4s^1^).
300	Most intense band; assigned to electronic transitions in the host matrix and to Pb^2+^ ions.
320–420	Charge transfers of Cu-O [21].
335	Cu-O nanoparticles.
450–480	Cu^1+^ → Cu^2+^ inter-electronic transitions.
740	Low-intensity band; assigned to d-type transitions from the coordination of Cu^2+^ ions; observed for x = 30 mol% CuO sample.
600–850	Electronic transitions due to the Cu^2+^ ion.

**Table 3:** Wavenumbers and band assignments of the photoluminescence (PL) bands of the xCuO·10Sb_2_O_3_·(90 − x)[4PbO_2_·Pb] recycled system where x = 0–30% CuO.

Wavenumbers (nm)	Assignments of PL bands
330–440	3p^1^ → 1s^0^ transitions of Pb^2+^ ions.
420, 520, 630	3d^9^ 4s^1^ → 3d^10^ electronic transitions of Cu^2+^ ions [21].
400, 455, 470	Excitonic transitions from different conduction band sub-levels of Cu^2+^ ions superposed with Cu^1+^ ion interactions.
470–483	Transitions due to vacancies, defects and interstitial sites of copper ions.
520	Transitions due to the presence of Cu-O charge transfers.

**Table 4:** The electrochemical parameters derived from voltammetry assessed following the first cycle scan: anodic and cathodic potential (E_pA_, E_pC_), half-wave potential (E_1/2_), peak current density (I_A_), formal redox potential (E_f_), peak-to-peak separation (ΔE), where E_f_ = (E_pA_ + E_pC_)/2 and ΔE = E_pA_ − E_pC_.

x [mol%]	E_pA_ [V]	E_pC_ [V]	E_½_·10^−1^ [V]	Peak Current Density, I_A_·10^−3^ [A/cm^2^]	E_f_ [V]	ΔE [mV]
5%	0.27	−0.17	1.50	6.32	0.05	440
10%	0.24	−0.13	1.63	6.15	0.065	370
20%	0.245	−0.23	1.71	6.02	0.005	475
30%	0.26	−0.10	1.72	5.75	0.08	360

## Data Availability

The original contributions presented in this study are included in the article. Further inquiries can be directed to the corresponding author.
